# Characteristics of the early innate response induced by the aerosolized Ad5-vectored COVID-19 vaccine

**DOI:** 10.1186/s43556-024-00232-9

**Published:** 2024-12-05

**Authors:** Wan-Ru Zheng, Jun-Yan Dan, Nan Huo, Zhe Zhang, Li-Hua Hou

**Affiliations:** 1 School of Medicine, Zhejiang University, Hangzhou, 310058 China; 2 Laboratory of Advanced Biotechnology, Beijing Institute of Biotechnology, Beijing, 100071 China

Dear Editor,

The early innate response is considered to play a significant role in shaping the adaptive immune response. In the context of inflammation and pathogen challenge, circulating monocytes can differentiate into macrophages or dendritic cells and thus contribute to cytokine secretion and antigen presentation [1]. Recent studies have indicated that intramuscular injection of an mRNA-based or Ad5-vectored COVID-19 vaccine can rapidly induce an innate response in the blood, which is closely linked to the antibody response [2]. While previous research has focused mostly on early monocyte responses to intramuscular COVID-19 vaccines, our understanding of the responses induced by mucosal vaccines remains limited.

To address this gap in knowledge, we applied a multi-omics approach to investigate immune responses in five individuals who received the inhaled Ad5-vectored COVID-19 vaccine (Ad5-nCoV-BA.1-IH). This approach included high-temporal resolution immune profiling of cytokines, immune cells, and antibody kinetics as well as single-cell transcriptome sequencing (Fig. S1). Our findings revealed a significant increase in the levels of neutralizing antibodies specific to wild-type RBD in serum on day 28 compared to day 0 (Fig. 1a). The antibody response peaked at 3 months post-vaccination and then slowly decreased at 6 months. Unlike intramuscular mRNA COVID-19 vaccines, this inhaled vaccine elicits a delayed peak antibody response [3]. We observed similar antibody response kinetics to different SARS-CoV-2 variants, including the Alpha, Beta, Delta, Omicron BA.1 and Omicron BA.5 variants. There was considerable variation in antibody response between individuals, with two participants showing only a slight increase in antibody levels. Additionally, the antibody titers against the Omicron variants were lower than those against the wild-type, Alpha and Beta variants at all time points, which can be attributed to immune imprinting.

**Fig. 1:**
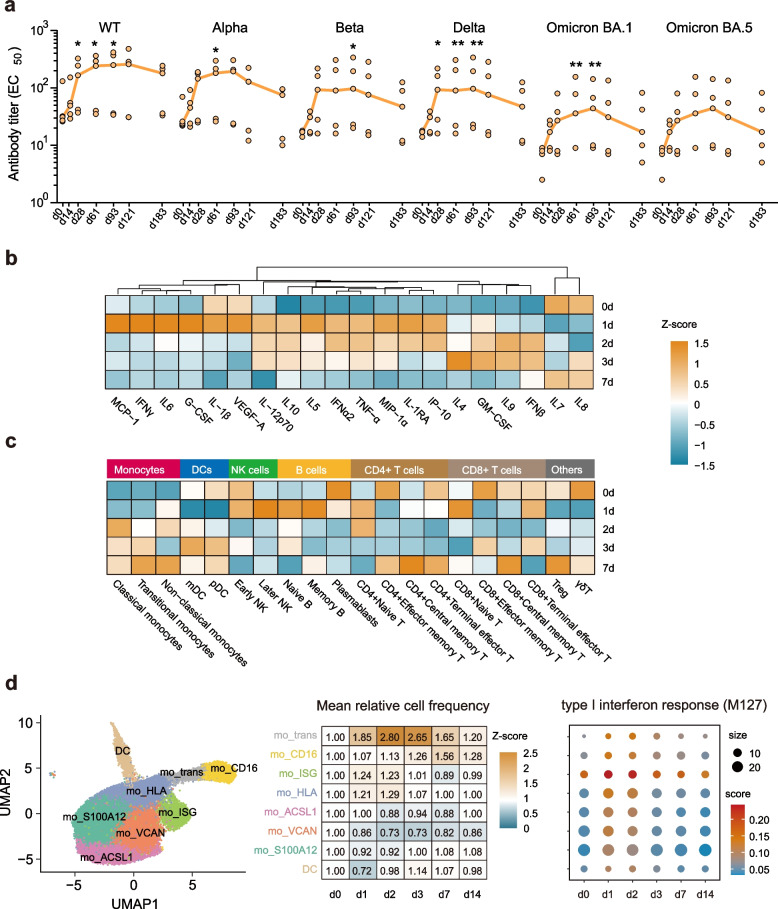
The early innate immune response to the aerosolized Ad5-vectored COVID-19 vaccine booster. **a** Neutralizing antibody responses against wild-type, Alpha, Beta, Delta, Omicron BA.1 and Omicron BA.5 SARS-CoV-2. Each orange dot represents one sample. The lines represents median. Statistical significance was determined by the Friedman test with Dunn’s multiple comparisons test. **b**-**c** Heatmaps depicting the dynamic changes in the levels of 20 cytokines (b) and 20 immune cells (c) on days 0, 1, 2, 3 and 7 after vaccination. **d** UMAP representation of 1 dendritic cell subpopulation and 7 monocyte subpopulations identified by single-cell transcriptional profiling (the left). Heatmap showing the fold changes in the frequencies of different monocyte subsets at each time point relative to day 0 (the middle). Heatmap showing the M127 pathway scores of different monocyte subsets at each time point (the right)

The early innate immune response was further characterized by analyzing the changes in 20 cytokines and 20 immune cell subsets in the peripheral blood. The plasma cytokine profile evaluated by Luminex assay revealed that the Ad5-nCoV-BA.1-IH booster triggered primarily interferon and inflammatory responses in the participants. Specifically, the IFNγ response was rapidly induced on day 1, followed by increase in IP-10, IFNα2 and IL-1RA, which were continuously activated on days 1–3 and returned to baseline levels on day 7 (Fig. 1b). These cytokines were reported to be transiently generated in recipients of an intramuscular mRNA COVID-19 vaccine on day 1, and they are strongly associated with monocyte and antibody response [4].

Further in-depth analysis of immune cells with 22 surface markers was conducted using CyTOF. The vaccine triggered variable immune cell responses on day 1, including increase in the numbers of NK cells, B cells, CD4 + Naïve T cells, and CD8 + Naïve T cells. While a distinct monocyte response was subsequently induced, characterized by a classical monocyte response with a delayed peak on day 2 and a continuously increasing transitional monocyte response (Fig. 1c). Together with the cytokine profile, these data led us to infer that this mucosal vaccine induces relatively slow and durable monocyte recruitment during the early phase of the innate immune response.

To gain a deeper understanding of the monocyte response induced by aerosolized Ad5-nCoV-BA.1-IH, single-cell cellular indexing of transcriptomes and epitopes by sequencing (CITE-seq) was performed on peripheral blood mononuclear cell (PBMC) samples from volunteers at 5 time points. A total of 122,522 high-quality cells were obtained and categorized into 19 distinct populations. Among these populations, monocytes exhibited 7 subpopulations, including mo_ISG (interferon-stimulated gene), mo_CD16, mo_trans, mo_HLA, mo_ACSL1, mo_VCAN and mo_S100A12, each with unique expression characteristics (Fig. 1d).

In line with the CyTOF findings, the vaccine-induced monocyte response was heightened during the first week after vaccination. The proportions of mo_trans, mo_CD16, mo_ISG, and mo_HLA increased significantly, with mo_trans peaking on days 2–3 and mo_CD16 peaking on day 7 after vaccination (Fig. 1d). In contrast, the frequencies of the remaining 3 monocyte subpopulations decreased to varying degrees. Given the important role of the interferon response in the immune response to mRNA-based and adenovirus-vectored COVID-19 vaccines [5], ISG expression in monocytes was further explored. The type I interferon response pathway (M127) and antiviral IFN signature (M75) were significantly upregulated in mo_ISG and other monocyte subpopulations (Fig. 1d). ISG activation peaked on days 1–2 in all monocyte subpopulations and lasted for 1 week in mo_trans and mo_CD16. This mucosal vaccine continuously activated mild innate immune and interferon responses for 7 days after vaccination, whereas these responses were temporarily but strongly induced in the recipients of the mRNA COVID-19 [4].

Our study has certain limitations. The first limitation is the limited number of clinical samples. While the innate immune response across different omics was consistent, the large variation between individuals prevented us from further analyzing the correlation between innate immune features and the specific antibody response. The second limitation is the absence of mRNA vaccine recipients in this study. The characterization of the innate immune response induced by the intramuscular mRNA COVID-19 vaccine that we describe is primarily based on the results of other studies. To address these limitations, future clinical studies should be carried out with a larger number of participants, including different vaccine subjects.

In summary, we conducted a systematic analysis of the immune responses in individuals who received the mucosal COVID-19 vaccine using multi-omics techniques. Our research reveals a significant increase in monocyte and interferon responses during the early stages following the administration of the vaccine. Compared with that to intramuscular mRNA vaccines, this response is more gradual and less intense, potentially impacting the dynamics of the antibody response. These findings provide important insights into the immune response mechanisms triggered by the mucosal COVID-19 vaccine in humans.

## Supplementary Information

Supplementary Material 1.

## Data Availability

The raw sequence data have been deposited in the Genome Sequence Archive in National Genomics Data Center (https://ngdc.cncb.ac.cn/gsa-human/browse/HRA007982). All other data are available in the main text or the supplemental information.
