# Supplementary material for: Characteristics of the early innate response induced by the aerosolized Ad5-vectored COVID-19 vaccine
Source: Mol Biomed. 2024 Dec 5;5:64. doi: 10.1186/s43556-024-00232-9 (PMC11618260; doi:10.1186/s43556-024-00232-9)
Supplement: Supplementary file 1 — Supplementary Material 1. [file 43556_2024_232_MOESM1_ESM.docx]

Supplementary Materials for

**Characteristics of the early innate response induced by the aerosolized Ad5-vectored COVID-19 vaccine**

Wan-Ru Zheng^1,2#^, Jun-Yan Dan^2#^, Nan Huo^2^, Zhe Zhang^2*^, Li-Hua Hou^1,2*^

^1^School of Medicine, Zhejiang University, Hangzhou 310058, China

^2^Laboratory of Advanced Biotechnology, Beijing Institute of Biotechnology, Beijing 100071, China

^#^These authors contributed equally

^*^Correspondence: [lxzzhe@126.com](mailto:lxzzhe@126.com) (Z.Z); [houlihua@sina.com](mailto:houlihua@sina.com) (L.H.)

**Materials**

**Study design**

Five healthy volunteers who had received 2 doses of intramuscular Ad5-nCoV (5×10^10^ viral particles) six months prior were chosen to receive 1×10^10^ viral particles of aerosolized omicron BA.1 Ad5-nCoV (Ad5-nCoV-BA.1-IH). None of the participants had previously been infected with SARS-CoV-2. PBMCs and plasma were collected on days 0, 1, 2, 3, 7 and 14 for analyses of the innate immune response. Serum samples were collected on days 0, 14, 28, 61, 93, 121 and 183 for analyses of the antibody response.

**Methods**

**RBD-ACE2 competitive binding assay**

RBD neutralizing antibodies against the different SARS-CoV-2 variants were assessed via a surrogate virus neutralization test using a commercial ELISA kit (Vazyme) as previously described(1). Serum samples were serially diluted by 3-fold from 1:5 to 1:1215 or 1:10935. Horseradish peroxidase-labeled recombinant RBD (HRP-RBD) (wild-type, Alpha, Beta, Delta, Omicron BA.1 or Omicron BA.5) was added to the diluted sera and incubated at 37°C for 30 min. HRP-RBD without serum was added in duplicate to each plate as the negative control. Subsequently, 100 μL of the dilution mixture was transferred to the corresponding wells of a microplate precoated with hACE2 protein. After an additional incubation at 37°C for 20 min, each well was washed 4 times with 350 μL of wash buffer. Then, 100 μL of TMB substrate was added, and the mixture was incubated for 15 min at 37°C. The reaction was stopped by adding 100 μL of stop solution, and the plate was immediately read at 450 nm. The antibody titer was calculated as the reciprocal of the dilution at which the OD value was reduced by 50% compared with that of the negative control (IC_50_) via nonlinear regression with four parameters in GraphPad Prism 9.3.1.

**Early innate immune response assay**

Multiple cytokine analysis was conducted using the MSD U-PLEX Viral Combo 1 assay (Meso Scale Discovery, MSD) in accordance with the manufacturer’s instructions. Briefly, 25 μL of serum or diluted standard was added to each well, and the plate were incubated at room temperature with shaking for 2 h. The plates were then washed, followed by the addition of 50 μL of detection antibody solution to each well. After a 1-h incubation, the plates were washed again, and 150 μL of MSD GOLD Read Buffer B was added to each well. The samples were then analyzed using an MSD instrument.

For CyTOF assay, immune cell analysis was performed via mass cytometry using the Maxpar Direct Immune Profiling Assay (Standard BioTools) according to the manufacturer’s instructions. Frozen PBMCs were thawed in a 37℃ water bath and gently resuspended in RPMI 1640 medium supplemented with 10% Fetal Bovine Serum (FBS) in conical tubes. The cells were washed with 5 mL of Maxpar Cell Staining Buffer (CSB). After cell counting, 3×10^6^ cells were incubated at room temperature for 10 min with Human TruStain FcX (BioLegend) in a 5 mL tube for each sample. The FcR-blocked cells were mixed with Maxpar CSB containing the antibody cocktail and incubated at room temperature for 30 min. Following another wash, the cells were fixed with a 1.6% formaldehyde solution at room temperature for 10 min. Finally, the cells were incubated with Cell-ID Intercalator-Ir at 4℃ overnight. The washed cells were resuspended in Milli-Q water supplemented with 1x EQ six element calibration beads (Standard BioTools), and 3×10^5^ cells were acquired on a Helios mass cytometer (Standard BioTools). The raw FCS files were normalized and concatenated using Fluidigm software. Gating and data analysis were performed using the Cytobank platform (Cytobank).

**scRNA-seq**

Frozen PBMCs were thawed as previously described. The cells were stained with Cell Hashing antibodies and flow cytometry antibodies against CD4, CD8, and CD19. Innate immune cells (CD4-CD8-CD19-) were isolated using an MA900 Cell Sorter (Sony) and were combined with total PBMCs at a 1:1 ratio. These cells were then loaded onto a 10X Chromium machine to construct 5' 10X libraries. Five samples from one time point were mixed onto one chip for sequencing. Approximately 25,000 cells were targeted for each chip. The 10x Genomics scRNA-Seq and TotalSeq-C libraries were pooled and sequenced on the Illumina Nova X Plus platform, resulting in 300G and 30G data, respectively. CellRangerv7.0 (10x Genomics) was used to demultiplex the raw sequencing data and quantify transcript levels against the 10x Genomics GRCh38-2020-A.

Single-cell RNA sequencing analysis was performed using Seurat V5.0. To detect samples contaminated with environmental mRNA or multiple cells, decontX V1.0(2) and scDblFind V1.16(3) were utilized for each chip, and cells with contamination >0.75 or scDblFinder score >0.75 were removed. Additionally, raw count data were filtered to eliminate cells with a mitochondrial RNA fraction exceeding 1% of the total RNA count per cell, and cells with fewer than 500 genes. We utilized the RPCAIntegration algorithm to remove batch differences and integrate data from different samples. TCR and BCR genes were excluded before further analysis because of their disruptive effects on cellular functional clustering. The first 30 principal components were employed for clustering and UMAP projections. Clusters were identified using the Louvain community algorithm at a resolution of 0.2. Subsequent analyses of monocytes and dendritic cells followed similar procedures, using the first 25 principal components and a resolution of 0.2. Pathway analysis was carried out using UCell and irGSEA, with reference genomes from blood transcription modules (BTMs)(4).

**Statistics analysis**

All data statistical analyses were performed using GraphPad Prism 9.3.1. or R 4.2.2. Multiple timepoint comparisons were analyzed by running a nonparametric (Friedman test) statistical test and corrected or uncorrected using Dunn test as indicated in the figure legends. Principal-component analysis was performed to evaluate the correlation and differences between samples on different time point using a permutational multivariate analysis of variance test based on Bray-Curtis distance measures. P values less than 0.05 were considered to indicate statistical significance.

**References**

1. Zhang Z, Wu S, Liu Y, Li K, Fan P, Song X, et al. Boosting with an aerosolized Ad5-nCoV elicited robust immune responses in inactivated COVID-19 vaccines recipients. Front Immunol. 2023;141239179. <https://doi.org/10.3389/fimmu.2023.1239179>.

2. Yang S, Corbett SE, Koga Y, Wang Z, Johnson WE, Yajima M, et al. Decontamination of ambient RNA in single-cell RNA-seq with DecontX. Genome Biol. 2020;21(1):57. <https://doi.org/10.1186/s13059-020-1950-6>.

3. Germain PL, Lun A, Garcia Meixide C, Macnair W, Robinson MD. Doublet identification in single-cell sequencing data using scDblFinder. F1000Res. 2021;10979. <https://doi.org/10.12688/f1000research.73600.2>.

4. Li S, Rouphael N, Duraisingham S, Romero-Steiner S, Presnell S, Davis C, et al. Molecular signatures of antibody responses derived from a systems biology study of five human vaccines. Nat Immunol. 2014;15(2):195-204. <https://doi.org/10.1038/ni.2789>.


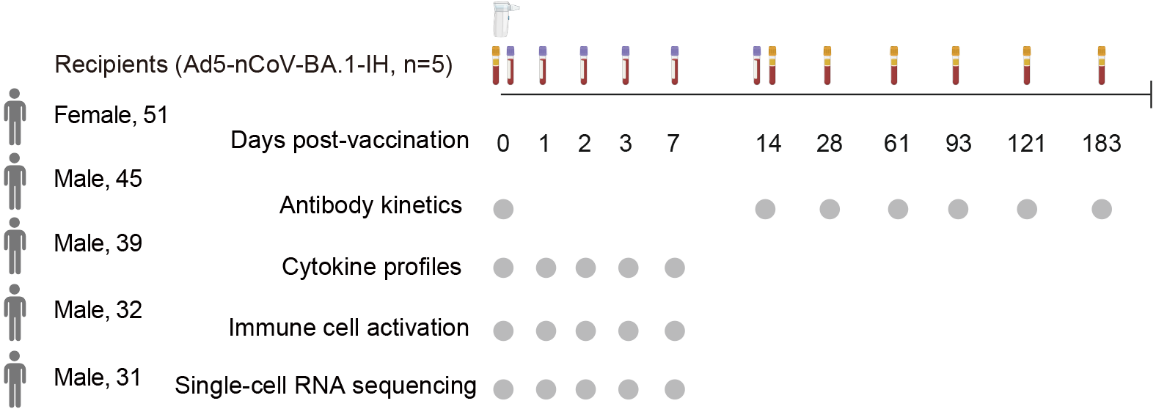
**Supplementary Figure**

**Supplementary Fig. S1** shows schematic representation of the study design. Five healthy volunteers were evaluated for antibody kinetics, cytokine profiles, immune cell activation and single-cell RNA sequencing at specific time points after vaccination.
